# Rapid reduction of suicidal ideation with transient dissociative and “drunken gait” symptoms after intranasal esketamine, with music intervention: a case report

**DOI:** 10.3389/fpsyt.2026.1739904

**Published:** 2026-04-30

**Authors:** Haihua Tian, Zongfeng Zhang, Yan He, Yuqiu Su, Xiaofeng Zhu, Yuhong Ding, Haihang Yu, Liuyin Jin, Jimeng Liu

**Affiliations:** 1 Department of Psychiatry, Affiliated Kangning Hospital of Ningbo University, Ningbo, Zhejiang, China; 2 Department of Psychiatry, Ningbo Kangning Hospital, Ningbo, Zhejiang, China; 3 The Second People’s Hospital of Lishui, Lishui, China

**Keywords:** dissociative symptoms, drunken gait, esketamine, music intervention, suicidal ideation, treatment-resistant depression

## Abstract

**Background:**

Treatment-resistant depression (TRD) is often accompanied by persistent suicidal ideation and poor response to conventional antidepressants. Intranasal esketamine, a non-selective NMDA receptor antagonist, provides rapid antidepressant and anti-suicidal effects; transient dissociation is common, whereas ataxia-like “drunken gait” is rarely described.

**Case presentation:**

A 19-year-old woman with recurrent depression and multiple prior suicide attempts received two courses of intranasal esketamine (56 and 84 mg, twice weekly for four weeks each). During the first course, she developed transient dissociative symptoms lasting approximately 55 minutes, predominantly characterized by depersonalization and accompanied by a “drunken gait. Under supervision, patient-selected music promptly reduced anxiety and disorientation, improving tolerability. From week 2, prophylactic oxazepam 25 mg prevented mild delayed dissociative episodes. The second course was uneventful. By the end of treatment, suicidal ideation had resolved.

**Discussion:**

This case illustrates rapid improvement of depressive symptoms and suicidality with esketamine and shows that dissociative reactions are typically self-limiting and manageable. Music intervention may attenuate these experiences by modulating prefrontal–amygdala circuits and autonomic regulation, thereby reducing anxiety and enhancing reality orientation. Individualized monitoring and psychological support are recommended components of esketamine services.

**Conclusion:**

Intranasal esketamine is a promising option for TRD with elevated suicide risk. Patient-selected music is a feasible adjunct to manage dissociative side effects and may improve safety and adherence. Further research should clarify mechanisms and long-term safety.

## 1 Introduction

Major depressive disorder (MDD) is a common psychiatric condition and one of the leading causes of disability worldwide, affecting approximately 264 million people (1). Among patients with MDD, about 30% show little or no response to two or more adequate antidepressant trials and are classified as having treatment-resistant depression (TRD) (2). Nevertheless, the terminology, definition, and clinical utility of TRD remain debated. Terms such as difficult-to-treat depression or multiple-treatment-failure depression have been proposed as potentially less stigmatizing alternatives. In addition, no clear boundary has been established between two, three, or more consecutive treatment failures, suggesting that treatment resistance may be better conceptualized as a continuum rather than a fixed threshold. Furthermore, evidence-based guidance for management after successive treatment failures remains limited, and current recommendations rely largely on expert consensus. Despite these conceptual challenges, TRD remains a prevalent and clinically significant condition, affecting 30%–40% of patients receiving antidepressant treatment and imposing a substantial burden on patients, caregivers, and society through increased disability and reduced quality of life (3). Given the limited efficacy of traditional monoaminergic antidepressants in TRD, novel therapeutic strategies targeting the glutamatergic, cholinergic, and opioid systems have emerged in recent years (4).

Intranasal esketamine, the S-enantiomer of ketamine, is a non-selective N-methyl-D-aspartate (NMDA) receptor antagonist (5). It has been approved in China as an adjunctive treatment for moderate-to-severe TRD. Esketamine’s major clinical advantage lies in its rapid onset of antidepressant effects and its capacity to rapidly alleviate suicidal ideation (6–8). Recent real-world evidence has also suggested possible gender-related differences in trajectories of suicidality and self-harming behavior following intranasal esketamine, indicating that demographic factors may be relevant when interpreting individual clinical responses, although current evidence remains preliminary (9).

However, esketamine’s central nervous system (CNS) side effects are relatively prominent, the most common being transient dissociative symptoms, including derealization, depersonalization, perceptual distortion, illusion, and hallucination (10). These symptoms typically occur within minutes after administration and resolve spontaneously within approximately 90 minutes. Repeated treatments have been reported to reduce the severity of such symptoms. Recently, several studies have explored non-pharmacological approaches—such as music intervention—to alleviate esketamine-associated anxiety, fear, and dissociative experiences, showing promising effects (11, 12).

To date, however, detailed case reports describing motor coordination–related dissociative symptoms, such as “drunken gait,” remain scarce. Drunken gait is characterized by transient balance disturbance, unsteady walking, and impaired coordination, potentially linked to esketamine-induced central dissociation and vestibular dysfunction.

Here, we report a case of recurrent depressive disorder in which the patient underwent two courses of intranasal esketamine treatment. During the first course, the patient experienced dissociative symptoms accompanied by a transient “drunken gait,” which markedly improved following music intervention. More broadly, music-based interventions have shown potential as adjunctive approaches in psychiatric care. Meta-analytic evidence suggests beneficial effects on depressive and anxiety symptoms and quality of life across several psychiatric conditions; however, the certainty of evidence is generally low, and their role in managing acute esketamine-related dissociative experiences has not yet been established (13). Notably, despite a history of multiple suicide attempts and persistent suicidal ideation, the patient’s suicidal thoughts significantly decreased after the second course of esketamine therapy. This case provides novel clinical evidence for motor-type dissociative reactions induced by esketamine and highlights the potential role of non-pharmacological interventions in their management.

## 2 Methods

### 2.1 Patient and diagnosis

This case involved a 19-year-old female patient whose depressive symptoms began in adolescence and had persisted for five years without systematic treatment before being admitted to Ningbo Kangning Hospital for comprehensive psychiatric evaluation and management. The patient met the diagnostic criteria for recurrent depressive disorder, current episode severe with psychotic symptoms according to the International Classification of Diseases, 10th Revision (ICD-10). Throughout the course of illness, she exhibited multiple episodes of self-harm and suicidal behavior. All diagnoses and treatment decisions were jointly confirmed by two board-certified psychiatrists with associate chief physician or higher qualifications. To improve diagnostic accuracy, the assessment included a detailed psychiatric interview, mental status examination, collateral history obtained from family members, longitudinal review of the illness course, and routine ancillary examinations when clinically indicated. Differential diagnosis focused particularly on trauma-related disorders, substance use-related conditions, and neurodevelopmental disorders. Although the patient had a long history of depressed mood and self-injurious behavior, she did not report core trauma-related symptoms such as intrusive re-experiencing, persistent avoidance, or hyperarousal, and there was insufficient evidence to support a trauma-related disorder. Both the patient and her family denied any history of alcohol misuse, illicit substance use, or misuse of psychoactive medications, and no temporal association was identified between symptom exacerbation and substance exposure; therefore, a substance-induced condition was considered unlikely. In addition, developmental history did not reveal persistent childhood-onset symptoms of inattention, hyperactivity/impulsivity, social communication deficits, restricted interests, or stereotyped behaviors, making a neurodevelopmental disorder less likely. Taken together, the longitudinal course, symptom profile, and clinical evaluation supported the diagnosis of severe recurrent depressive disorder with psychotic symptoms.

### 2.2 Ethical statement

This report was based on clinical observational data. Ethical approval for this case report was obtained from the Ethics Committee of Ningbo Kangning Hospital (Approval No. NBKNYY-2025-LC-88). Written informed consent for the use of anonymized clinical data for research and publication was obtained from the patient. The music-listening measure described in this report was used as a supportive non-pharmacological adjunct during clinical care rather than as a prespecified research intervention.

### 2.3 Assessment tools and monitoring

During different treatment stages, standardized clinical scales were used for serial assessments: 1.Hamilton Depression Rating Scale (HAMD-24): to evaluate the severity of depressive symptoms; 2.Hamilton Anxiety Rating Scale (HAMA): to assess anxiety levels; 3.Brief Psychiatric Rating Scale (BPRS): to monitor psychotic features, including hallucinations, delusions, and emotional agitation. In addition, vital signs (blood pressure, heart rate, and oxygen saturation) were continuously monitored during treatment, and drug tolerability and adverse reactions were systematically recorded.

### 2.4 Treatment protocol

Between June 2024 and June 2025, the patient received comprehensive treatment including: 1.Antidepressants: selective serotonin reuptake inhibitors (SSRIs, e.g., fluoxetine, paroxetine) and serotonin–norepinephrine reuptake inhibitors (SNRIs, e.g., venlafaxine); 2.Mood stabilizers: lithium carbonate, sodium valproate, and lamotrigine; 3.Antipsychotics: quetiapine and paliperidone, prescribed for hallucinations and behavioral impulsivity;4.Physical therapy: modified electroconvulsive therapy (MECT). Despite multiple pharmacological and physical interventions, the patient continued to experience persistent suicidal ideation and impulsive self-harm behavior. On January 3, 2025, she commenced intranasal esketamine treatment under close medical supervision. The initial course involved 56 mg per session, administered twice weekly for four weeks (eight sessions total). Vital signs and clinical responses were continuously monitored during administration.

During the first administration, the patient developed prominent dissociative symptoms, mainly manifested as depersonalization, accompanied by transient gait instability clinically described as a “drunken gait.” To alleviate her distress, a music-listening procedure with a relatively fixed implementation process was introduced under direct medical supervision as a supportive non-pharmacological measure. The patient was instructed to listen to self-selected music that she perceived as familiar and soothing.

During the second treatment session, the patient expressed anxiety about the recurrence of dissociative symptoms and voluntarily requested to listen to self-selected music as a means of distraction. Music was initiated approximately 15 minutes after intranasal esketamine administration and lasted for about 30–40 minutes. Within several minutes of listening, the patient’s emotional state appeared more stable than before, and her anxiety and disorientation were alleviated to some extent. Based on this clinical observation, the same music-listening procedure was adopted in all subsequent treatment sessions and documented as a supportive non-pharmacological measure.

In subsequent sessions, the procedure was implemented in a generally consistent manner: it was usually initiated approximately 15 minutes after esketamine administration, when dissociative discomfort was more likely to emerge, and continued for approximately 30–40 minutes. Music was played in a quiet clinical environment at a comfortable low-to-moderate volume and was adjusted according to the patient’s tolerance at the time. No prespecified music genre was required; rather, music selection was primarily based on the patient’s personal preference and acceptability in order to minimize distress and maintain engagement. Changes in subjective distress, dissociative symptoms, gait stability, and overall tolerability were assessed mainly through bedside clinical observation and patient self-report, without the use of formal rating scales.

Overall, as treatment progressed, the patient’s general tolerability of the treatment process appeared to improve. In this case, the music-listening procedure was documented as a supportive non-pharmacological measure rather than a standardized treatment protocol validated for efficacy.

A second course of intranasal esketamine treatment was initiated on June 23, 2025, with an increased dose of 84 mg per session, administered twice weekly for four weeks. Adverse reactions were systematically recorded throughout both treatment phases.

## 3 Case presentation

### 3.1 Clinical course and previous treatments

The patient was a 19-year-old female who first developed depressive symptoms at the age of 14, characterized by persistent low mood, anhedonia, insomnia, excessive rumination, tearfulness, fatigue, and impaired concentration, accompanied by marked academic decline and social withdrawal. The symptoms progressively worsened, leading to recurrent suicidal ideation and self-injurious behaviors. She had no history of systematic treatment, no family history of psychiatric disorders, and no substance use.

In the initial five years of illness, the patient did not receive any pharmacological or psychological intervention. In March 2023, she presented to the emergency department following wrist-cutting behavior and was diagnosed with depressive state. Psychotherapy was initiated but yielded poor outcomes. Since 2024, she had been receiving systematic antidepressant treatment at Ningbo Kangning Hospital, including multiple classes of antidepressants—SSRIs, SNRIs, tricyclic antidepressants, and monoamine oxidase inhibitors—partly in combination with mood stabilizers (lithium, valproate, lamotrigine) and antipsychotics (**Table 1**). Cognitive behavioral therapy (CBT) was also implemented.

**Table 1:** Summary of adverse reactions and clinical course during the first esketamine treatment phase (January 3–21, 2025).

No.	Date (2025)	Description of adverse reaction	Emotional/mental state after administration	Outcome
1	3-Jan	About 2 min after nasal spray: unsteady sitting, leg weakness, involuntary limb movements, and euphoria (“drunken-like” state). Required assistance to bed; gradually calmed after ~1 h.	Evening mood stable; transient impulsivity and self-biting episode next day; mild headache and gait instability persisted.	Recovered within 1 h
2	7-Jan	Immediate excitation, limb tremor, and weakness; unable to control movements. Calmed with soothing music; consciousness normalized after ~1 h.	Reported “light and floating happiness” the next morning; mild fatigue and headache.	Recovered within 1 h
3	10-Jan	Unsteady gait with strong urge to walk; severe nausea and vomiting; marked headache; verbalized “too uncomfortable to continue.”	Emotional fluctuation later that night, transient irritability, self-injury (hand scratching), stabilized next day.	Recovered within 1 h
4	14-Jan	Bitter taste, dizziness, nausea, vomiting, confusion, speech slowness, and depersonalization (“Who am I? Where am I?”). Needed supervision and reassurance; symptoms resolved in ~40 min.	Reported mood improvement (“like anesthesia”) and good sleep afterwards.	Recovered within 1 h
5	17-Jan	Irritability and physical agitation after dosing; required brief restraint; headache and nausea relieved by sugar intake.	Later mood elevated and energetic; transient low mood on Jan 19 without self-harm.	Recovered within 1 h
6	21-Jan	Sudden weakness and collapse after spray; slurred speech, depersonalization (“tongue not my own”), nausea, and confusion. Recovered in ~40 min.	Mild transient emotional blunting; stable mood next day.	Recovered within 1 h

Adverse events were observed during the first esketamine treatment phase (56 mg twice weekly for 4 weeks). All reactions were transient, self-limited, and resolved within one hour under clinical supervision. No severe medical complications occurred. Music listening was introduced from the second administration onward as a non-pharmacological strategy to alleviate dissociative symptoms.

After 20 sessions of repetitive transcranial magnetic stimulation (rTMS) failed to produce significant improvement, the patient underwent nearly 30 sessions of modified electroconvulsive therapy (MECT), which produced only transient relief of anxiety symptoms. Subsequently, she experienced several suicide attempts via drug overdose (including fluoxetine, lorazepam, and quetiapine), necessitating repeated hospitalizations for stabilization.

**Figure 1:**
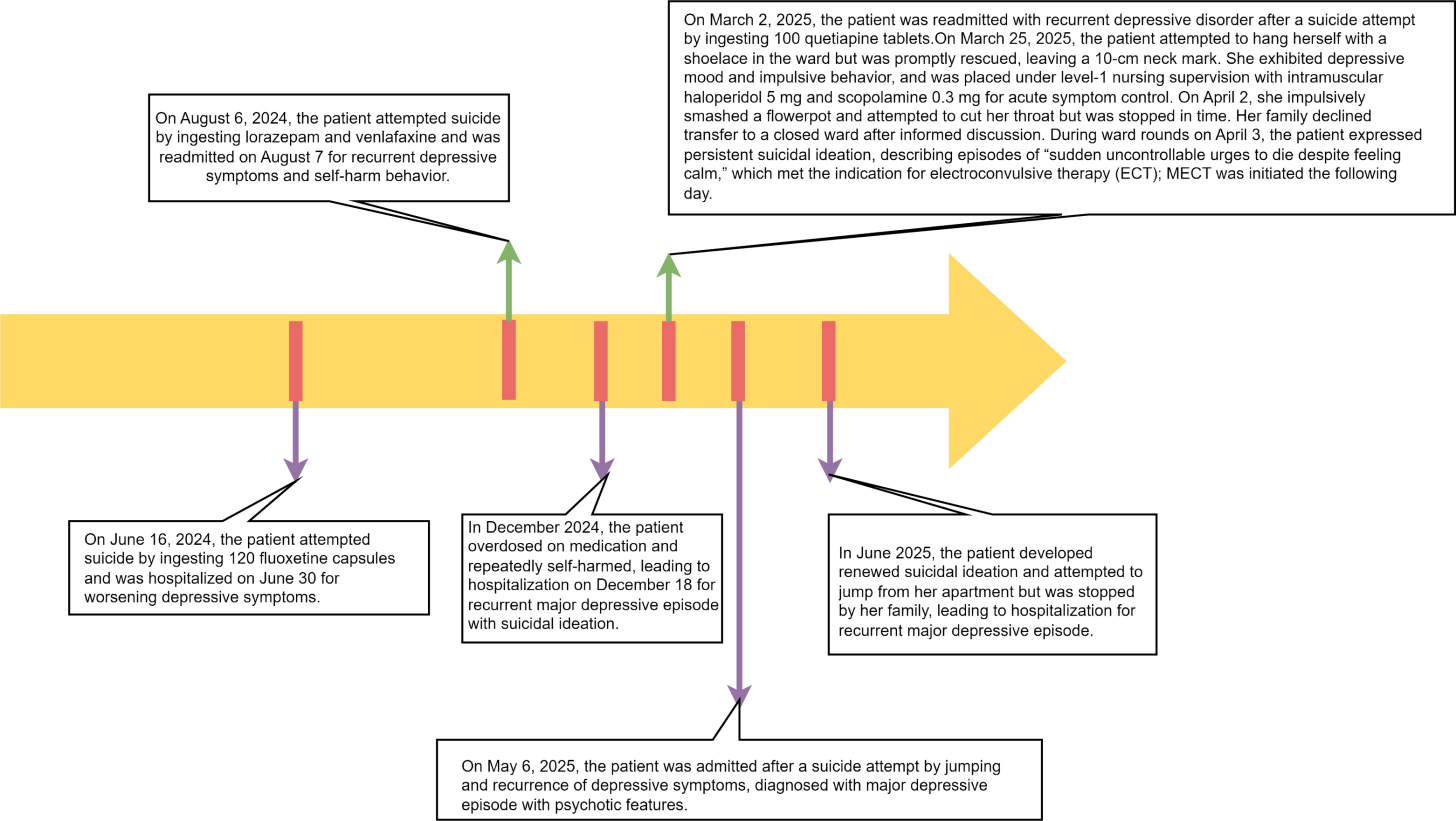
Timeline of the patient’s suicidal behaviors and hospitalizations (June 2024–June 2025).

**Figure 2:**
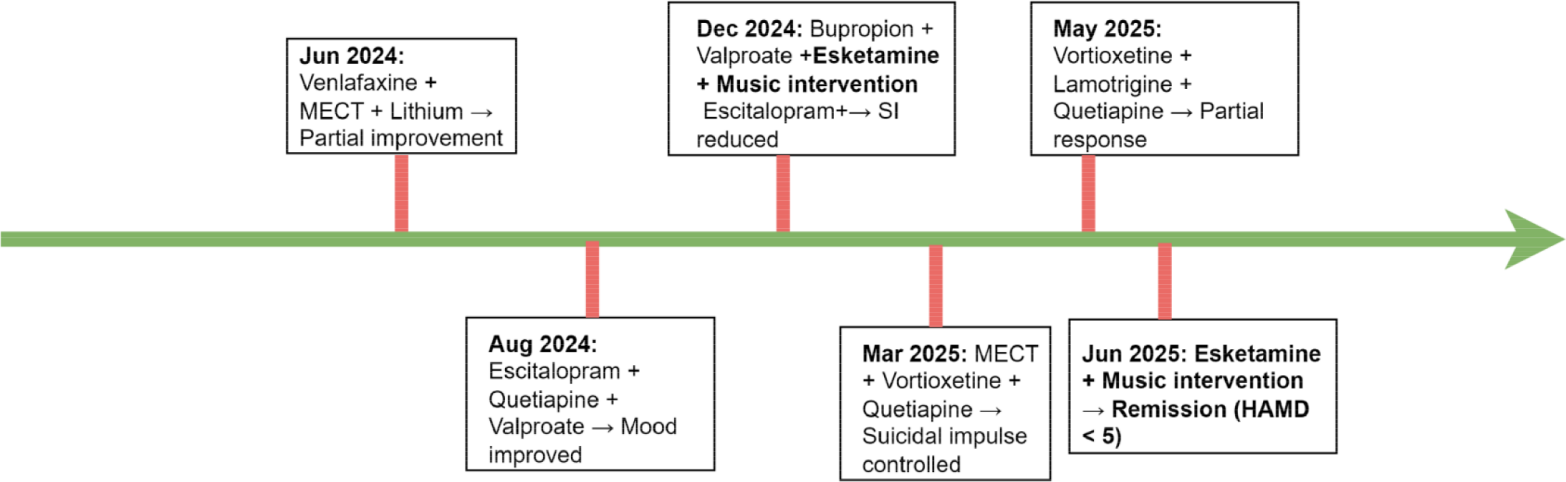
Timeline of the patient’s major pharmacological and non-pharmacological interventions (June 2024–June 2025).

**Figures 1** and **2** illustrate the patient’s seven-year clinical trajectory, six hospitalizations, and the timeline of major therapeutic interventions.

### 3.2 Esketamine treatment, dissociative symptoms, and management

On January 3, 2025, during her third hospitalization, the patient initiated intranasal esketamine treatment. The first course consisted of 56 mg per session, administered twice weekly for four weeks (a total of eight sessions). The second course, beginning on June 23, 2025, involved an increased dosage of 84 mg per session, also administered twice weekly for four weeks.

During the second treatment session, the patient expressed anxiety about the recurrence of dissociative symptoms and voluntarily requested to listen to self-selected music as a means of distraction. Music was initiated approximately 15 minutes after administration and continued for about 30–40 minutes. Within several minutes of listening, the patient’s emotional state appeared more stable than before, and her anxiety and disorientation were alleviated to some extent. Thereafter, the same music-listening measure was used in subsequent sessions and documented as a supportive non-pharmacological adjunctive measure. Overall, this measure appeared to be associated with reduced subjective distress and greater emotional stability in some sessions; however, acute adverse reactions remained variable across treatments, and relatively severe discomfort still occurred in individual sessions. Therefore, any improvement in overall tolerability should be interpreted with caution.

Notably, although the music-listening measure was introduced from the second treatment session onward, the patient still experienced marked gait instability, nausea and vomiting, and severe headache during the treatment on January 10, 2025. This suggests that the measure may have been more relevant to alleviating subjective anxiety, distress, and disorientation than to consistently preventing all acute esketamine-related adverse effects.

Given that the patient developed only delayed anxiety and dissociation-related discomfort after intranasal esketamine treatment, and considering that concomitant use of esketamine and benzodiazepines may increase the risk of sedation, low-dose oxazepam was selected in this case as a short-term supportive measure based on clinical judgment. Compared with some long-acting benzodiazepines, oxazepam undergoes direct glucuronidation and has no active metabolites, making its pharmacokinetic profile relatively more predictable and therefore more suitable for the adjunctive management of such brief and fluctuating symptoms. During treatment, vital signs, including blood pressure, heart rate, and oxygen saturation, remained generally stable. As intranasal esketamine treatment progressed, the patient’s suicidal ideation gradually decreased, and no further self-harming or suicidal behaviors were observed. Her mood, sleep, and social functioning also improved progressively and remained relatively stable during follow-up.

**Figure 3:**
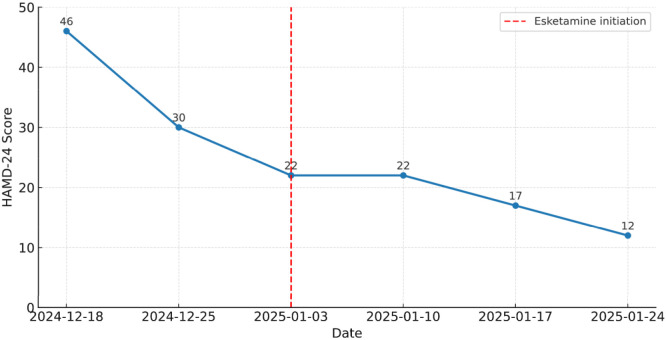
Changes in the patient’s HAMD-24 scores during the first course of intranasal esketamine treatment (December 2024–January 2025).

**Table 2** provides a quantitative summary of the changes in HAMD-24 scores across treatment. The score decreased from 46 at baseline on December 18, 2024 to 30 on December 25, 2024, representing a 34.8% reduction from baseline. At the time of esketamine initiation on January 3, 2025, the HAMD-24 score had further decreased to 22, corresponding to a 52.2% reduction from baseline and indicating response-level improvement under prior treatment. The score remained at 22 on January 10, 2025, and then declined further to 17 on January 17, 2025 and 12 on January 24, 2025, representing 63.0% and 73.9% reductions from baseline, respectively. **Figure 3** illustrates this downward trajectory visually, showing that depressive symptoms had already improved before esketamine initiation but continued to decline thereafter.

**Table 2:** Changes in HAMD-24 scores across treatment.

Date	HAMD-24 score	Change from baseline	% reduction from baseline
2024-12-18	46	0	0%
2024-12-25	30	-16	34.8%
2025-01-03	22	-24	52.2%
2025-01-10	22	-24	52.2%
2025-01-17	17	-29	63.0%
2025-01-24	12	-34	73.9%

From the seventh session of the first course onward, the patient no longer experienced significant adverse reactions, and no adverse effects were observed during the second course. By the end of the second treatment course, her HAMD-24 score had decreased to 5, meeting the criterion for depressive remission. The primary goal of the second course—to further attenuate suicidal ideation and impulsive behavior—was successfully achieved. The patient continues to attend regular outpatient follow-ups, maintains good medication adherence, and remains emotionally stable with sustained remission.

**Table 1** summarizes the adverse events observed during the first esketamine treatment course, while **Table 2** and **Figure 3** together demonstrate the progressive reduction in depressive symptom severity across the treatment period.

## 4 Discussion

We report a case of a 19-year-old female patient with TRD who participated in a clinical trial evaluating the efficacy and safety of intranasal esketamine. The patient received intranasal esketamine twice weekly for four weeks at doses of either 56 mg or 84 mg per session. On the first treatment day, she received two nasal spray devices (28 mg per device, total 56 mg). Approximately two minutes after the initial spray, she developed a transient episode of *“drunken gait”* lasting for about one hour. The patient was encouraged to listen to music during treatment, which significantly alleviated her symptoms. Listening to personally preferred music immediately after administration, along with reassurance from medical staff, appeared to help control the dissociative reaction.

Intranasal esketamine has been shown to be effective in patients with suicidal ideation, and due to its rapid antidepressant onset—sometimes within a single dose—it is being actively explored for its potential benefits in individuals with active or planned suicidal intent (14, 15). In our case, the patient’s depressive symptoms improved rapidly and were sustained over the following days. Notably, suicidal ideation was no longer reported in subsequent follow-ups.

The precise mechanism underlying ketamine’s antidepressant and anti-suicidal effects remains unclear. Proposed mechanisms include NMDA receptor antagonism, AMPA receptor activation, anterior cingulate cortex activation, increased connectivity between the insula and the default mode network (DMN), and enhancement of neurovascular endothelial growth factor signaling (16). Several studies have demonstrated that ketamine enhances neuroplasticity by stimulating brain-derived neurotrophic factor (BDNF) production and activating the mammalian target of rapamycin (mTOR) signaling pathway (17). Regulation of the mTOR pathway promotes additional BDNF expression, thereby increasing synaptic plasticity and dendritic growth in brain regions involved in emotion regulation and mood control. Compared with oral antidepressants, esketamine may exert a more direct stimulatory effect on the BDNF–mTOR axis, which could explain both its rapid onset and its sustained efficacy even after drug elimination.

Concerns regarding the safety of esketamine mainly involve its potential for misuse, tolerance, and withdrawal, underscoring the need for administration strictly under medical supervision in a controlled clinical setting. Long-term use may be associated with cognitive deficits and working memory impairment; however, current data on such long-term effects remain limited (18). These adverse cognitive outcomes are primarily observed among individuals with ketamine abuse, and have not been reported under supervised therapeutic use. The most common adverse effects of esketamine include sedation, nausea, mild dissociation (feeling detached or unreal), dizziness, and headache. Slight elevations in blood pressure and mild respiratory depression can occur, requiring close monitoring. In rare cases, moderate to severe dissociative phenomena similar to those induced by high-dose ketamine may appear, though such reactions are infrequent and dose-dependent (19).

The cerebellum plays a critical role in maintaining motor balance and movement coordination, particularly in gait, posture, and complex motor tasks. Dysfunction of the cerebellum may result in ataxia and a *“drunken”* or unsteady gait. Given its compact structure but high neuronal density, primarily within the cerebellar cortex (20), transient cerebellar dysregulation induced by esketamine-related NMDA modulation might contribute to such symptoms. Another possible explanation is that, following initial exposure to esketamine, the central nervous system may become transiently susceptible to spontaneous dissociative experiences, resulting in brief episodes predominantly characterized by depersonalization, resembling a transient dissociative state. In addition, the phenomenological framework proposed by Sarasso et al. (2024) suggests that esketamine-related experiences may involve disembodiment and alterations in affective resonance. This provides an alternative perspective for understanding the atypical dissociative symptom of “drunken gait” observed in this case, suggesting that it may reflect not only impaired motor coordination but also a transient disruption of bodily self-experience (21).

Interestingly, in this case, the music-listening measure was temporally associated with a reduction in dissociation-related discomfort and anxiety symptoms. Previous systematic reviews and meta-analyses have suggested that music-based interventions may have adjunctive value in psychiatric treatment and may be associated with improvements in depressive symptoms, quality of life, and certain anxiety-related outcomes (22), although the overall quality of the available evidence remains limited (13, 23). Theoretically, music may exert a supportive effect by enhancing emotional regulation, reducing subjective distress, and improving the overall treatment environment and experience; however, sufficient evidence is still lacking regarding the specific neurobiological mechanisms underlying its role in esketamine-related dissociative symptoms (12, 24). Therefore, the music measure used in this case should be regarded as a supportive non-pharmacological adjunct with potential clinical feasibility, and its actual effects still require further validation in larger-sample prospective studies.

In addition, the patient did not experience further dissociative symptoms or “drunken gait” during the second treatment course. This finding is consistent with previous reports suggesting that such adverse reactions may lessen with repeated administration; however, given that the dosage was higher during the second course, and that environmental support, individual adaptation, and the natural fluctuation of symptoms may also have contributed in this case, it would not be appropriate to attribute this change simply to “drug tolerance.” Her suicidal behavior also ceased completely, suggesting that intranasal esketamine was associated with sustained therapeutic benefit in this case.

This clinical observation highlights three potentially important implications:

(1) Intranasal esketamine may be an effective therapeutic option for TRD patients with suicidal ideation; (2) Dissociative symptoms, though common, are generally reversible—environmental reassurance and music intervention can significantly enhance tolerability and compliance; (3) Individualized monitoring and multimodal psychological support should be integral components of esketamine treatment protocols.

Several limitations should be acknowledged. First, this is a single-patient case report, and the observations are inherently individual-specific, limiting generalizability. Second, in the absence of any control or comparison condition, the relative contributions of esketamine, music listening, environmental reassurance, cumulative effects of prior treatments, and the natural course of illness cannot be determined. Third, the observed effects of the music-listening measure may have been influenced by placebo effects, treatment context, staff support, and patient expectations, and therefore should be interpreted cautiously. Finally, the music-listening measure used in this case was a preference-based supportive strategy rather than a standardized and validated intervention, which limits its reproducibility and broader applicability. Future larger-scale prospective studies are needed to verify these observations and to further explore the underlying mechanisms and potential clinical applicability.

## 5 Conclusion

This case highlights that intranasal esketamine, as a rapidly acting antidepressant, can not only produce a marked improvement in depressive symptoms in patients with TRD but also effectively control persistent suicidal ideation and impulsive behavior within a short period. Although dissociative symptoms predominantly characterized by depersonalization may occur, accompanied by manifestations such as a “drunken gait,” these symptoms are usually transient and self-limiting. Appropriate psychological reassurance and music intervention can substantially enhance treatment tolerability and adherence. With continued treatment, the patient’s tolerability improved, symptoms remitted and remained stable, and no further adverse events were observed.

This case underscores the potential clinical value of esketamine for TRD patients with high suicide risk and proposes music intervention as a feasible adjunctive strategy for managing dissociative reactions. Further systematic studies are warranted to elucidate its underlying neurobiological mechanisms and long-term safety.

## 6 Patient perspective

The patient reported that intranasal esketamine treatment helped reduce her suicidal thoughts and alleviate depressive symptoms. She acknowledged experiencing mild nausea and headache during one session. Overall, she found the treatment highly beneficial and stated that it significantly improved her quality of life.

## Data Availability

The raw data supporting the conclusions of this article will be made available by the authors, without undue reservation.
